# Tissue Factor-inhibited Beclin1-autophagy via BCL2 overexpression suppresses the differentiation of human monocytes into mature osteoclasts

**DOI:** 10.1590/1678-4685-GMB-2025-0028

**Published:** 2026-05-11

**Authors:** Lihua Wu, Tingwei Gao, Dianshan Ke, Guoming Liu

**Affiliations:** 1 Fujian Medical University ShengLi Clinical College, Fujian Provincial Hospital, Fuzhou University Affiliated Provincial Hospital, Department of Otolaryngology , Fuzhou, Fujian, China.; 2 Fujian Medical University ShengLi Clinical College, Fujian Provincial Hospital, Fuzhou University Affiliated Provincial Hospital, Department of Orthopedics, Fuzhou, Fujian, China.

**Keywords:** Tissue Factor, osteoclast, monocyte, bcl2, autophagy

## Abstract

Tissue Factor (TF) can inhibit osteoclastogenesis. Moderate autophagy is an essential factor for osteoclastogenesis. As a molecule present in human monocytes, TF can inhibit autophagic activity through B-cell lymphoma-2 (BCL2) overexpression; this suggests that the anti-osteoclastogenic role of TF may occur in human monocytes and be associated with BCL2-dependent autophagy. Our study aimed to explore the relationship between TF-regulated BCL2-autophagy signaling and osteoclast-differentiation fate of human monocytes. Cell enrichment based on fluorescence-activated cell sorting (FACS) was used to enrich TF-associated monocytic clusters. Then, a series of functional assays were performed to investigate the roles of TF in monocytic parameters associated with osteoclast precursors (OCPs). The results showed that under osteoclastic induction, the osteoclastogenic ability and autophagic activity in enriched TF^-^ monocytes were enhanced whereas TF^+^ monocytes exhibited opposite results. BCL2 expression and the interaction between BCL2-Beclin1 in TF^-^ monocytes decreased while those of TF^+^ monocytes were contrary. The osteoclastogenic ability of TF^-^ monocytes decreased again with autophagy inhibition with Beclin1 underexpression. The osteoclastogenic ability of TF^+^ monocytes increased again with enhanced autophagy with BCL2 inhibition and Beclin1 overexpression. Overall, TF increases BCL2-Beclin1 stability by upregulating BCL2 expression, thereby decreasing Beclin1-dependent autophagy and subsequent monocytic osteoclastogenesis in human monocytes.

## Introduction

The dynamic balance between bone resorption dominated by osteoclasts and bone formation dominated by osteoblasts maintains normal bone remodeling (Wang et al., 2022). Excessive osteoclastogenesis can disrupt the above balance; this leads to the transformation of normal bone remodeling into enhanced bone resorption, thereby resulting in pathological bone destruction, including osteoporosis and auditory ossicular resorption (Kanzaki et al., 2006; Bolamperti et al., 2022; Song et al., 2022). As a serious health issue that poses a threat to socio-economic development, the resolution of pathological bone destruction has become a major challenge for orthopedics and related disciplines. Primary osteoporosis caused by menopause, as well as various pathological bone losses are all associated with excessive osteoclastic bone resorption (Kanzaki *et al*., 2006; Charles and Aliprantis, 2014; Fu et al., 2023; Cui et al., 2024; Ke et al., 2024; Li et al., 2024). Therefore, osteoclasts are important biological targets to address the aforementioned challenges. In the past, a large amount of research on osteoclasts has focused on mature cells originating from mouse bone marrow macrophages (BMMs), neglecting human osteoclast precursors (OCPs). Previous studies demonstrated that human monocytes can serve as human osteoclast precursors (OCPs) and possess excellent potential for osteoclastogenesis (Hansen et al., 2024; Moura et al., 2024). However, the underlying mechanism by which human monocytes differentiate into mature osteoclasts is still unclear at present.

To clarify osteoclast-differentiation fate of human monocytes, we need to conduct a detailed exploration based on a novel molecule that bridges monocytes and osteoclastogenesis. Abundant literatures report that tissue factor (TF) is highly expressed in human monocytes (Grover and Mackman, 2018; Musgrave et al., 2023; Olivieri et al., 2023). Moreover, the inhibitory effect of TF on osteoclastogenesis is supported by relevant research (Ehara et al., 2021). However, the role of TF in the differentiation of human monocytes into mature osteoclasts has not been elucidated. As a powerful intracellular protective mechanism, autophagy is beneficial for osteoclast formation and bone resorption activity (Montaseri et al., 2020; Wang et al., 2023). Furthermore, the autophagy-inhibiting property of TF was revealed by several studies (Deng et al., 2016; Huang et al., 2017; Liu et al., 2022). In addition, TF is a promoter for B-cell lymphoma-2 (BCL2) overexpression (Fang et al., 2008; Wu et al., 2016). Beclin1, as the main autophagy regulatory molecule, maintains the silence of autophagic flux by binding to BCL2 while activating type-III phosphoinositide 3-kinase (PI3KC3) after dissociating from BCL2-Beclin1 complex, thereby promoting autophagic responses, and high expression of BCL2 inhibits autophagy by preventing the dissociation of BCL2-Beclin1 complex (Pattingre et al., 2005). In summary, we speculate that TF enhances the stability of BCL2-Beclin1 complex through BCL2 overexpression, thereby weakening the release of Beclin1 and Beclin1-related autophagic activity; this blocks the differentiation of human monocytes into mature osteoclasts.

Our objective is to uncover a novel biological phenomenon and its underlying mechanisms through a series of in vitro studies, which elaborates the significance of TF-regulated BCL2-autophagy signaling for monocytic osteoclastogenesis, thereby providing more clues for the treatment of osteoporosis caused by human-sourced osteoclasts.

## Material and Methods

### Ethics approval

Ethical approval for collection of human samples (Ethical Committee K2022-09-055) was provided by the Ethical Committee of Fujian provincial hospital, Fuzhou, China. Written informed consent was obtained from 5 participating probands prior to sample collection. Venous blood samples (5 mL per proband) were collected into the dedicated separation tubes (BD Vacutainer^®^ CPT™ Cell Preparation Tube, 362761, BD Biosciences; NJ, USA) via peripheral venipuncture from the antecubital vein; samples were processed immediately.

### Cell enrichments

The blood samples in the tubes were centrifuged at 1500g for 30 min at 20 °C to obtain peripheral blood mononuclear cells (PBMCs). PBMCs were incubated with CD3 (130-050-101, Miltenyi), CD19 (130-050-301, Miltenyi) MicroBeads and NKp46 (CD335)-coupled anti-Biotin MicroBeads (130-125-207/130-090-485, Miltenyi) on LS column (130-042-401, Miltenyi) for MicroBead sorting, which aimed to deplete mode. Subsequently, after blocking non-specific binding using Fc-Receptor Blocking Solution (Human TruStain FcX™, BioLegend, San Diego, CA, USA), cells were resuspended in Dulbecco’s Phosphate-Buffered Sallines (DPBS) along with 10% bovine serum albumin (BSA) for FACS, and stained for CD14-APC (301808, Biolegend), CD16-FITC (360716, Biolegend) for 20 minutes on ice. Single cell populations are classified based on the expression of CD14/CD16 within CD14^+^ gate relying on BD FACSAria III (BD Biosciences, NJ, USA). CD14^+^CD16^-^CD3^-^CD19^-^NKp46^-^ cells were enriched as CD14^+^CD16^-^ monocytes. Ultimately, the sorted cells were stained for TF-PE (365203, Biolegend) antibodies to enrich TF^+^ cells and TF^-^ cells. Further sorted cells (CD14^+^TF^+^ cells and CD14^+^TF^-^ cells) and control cells (CD14^+^ cells) are prepared for subsequent assays. 

### Osteoclastogenic assays

Enriched monocytes (96-well plates, 10000 cells/well) were cultured in complete α-Minimum Essential Medium (MEM) (containing 10% FBS, penicillin, streptomycin and L-glutamine) with 25 ng/mL M-CSF (Novoprotein, Jiangsu, China) plus 50 ng/mL RANKL (Novoprotein) for 7 days to induce human osteoclasts. After differentiation, osteoclastogenic ability was evaluated by Tartrate-Resistant Acid Phosphatase (TRAP) staining using related kit (BC5405, Solarbio; Beijing, China) according to manufacturer’s protocols. TRAP-positive multinucleate cells (more than 3 nuclei) were considered mature osteoclasts, and mature osteoclasts from each well are counted using the microscope for comparison between each group.

### Western-blot assays

Cells were treated into lysis buffer supplemented with phosphatase and protease inhibitors, and protein concentration of lysates was quantified. Equal amounts of denatured lysates were subjected to SDS-PAGE (80 V for stacking gel, 120 V for separating gel), followed by wet transfer of proteins to polyvinylidene fluoride membranes (PVDFs) at 100 V for 90 min on ice. After blocking with 5% milk in TBST for 1 hour at room temperature, PVDFs were combined with GAPDH (1:20000, 10494-1-AP, Proteintech; IL, USA), TF (1:1000, 28005-1-AP, Proteintech), LC3 (1:2500, 14600-1-AP, Proteintech), p62 (1:4000, 31403-1-AP, Proteintech), BCL2 (1:6000, 12789-1-AP, Proteintech), Beclin1 (1:6000, 11306-1-AP, Proteintech), BAX (1:1000, A12009, ABclonal) and PARP (1:1000, A19596, ABclonal) at 4 ℃ overnight. Then, PVDFs were incubated with HRP-conjugated goat anti-rabbit secondary antibody at 37 ℃ for 1 hour. Ultimately, the signals were observed using a Chemiluminescence Kit (Epizyme Biomedical Technology; Shanghai, China) and automatic digital gel/chemiluminescence image analysis system (4600SF, Tanon, China).

### Cell proliferation assays

Cell counting Kit-8 (CCK-8) analyses were performed to measure cell-proliferation capacity using the related kit (Dojindo, Kumamoto, Japan). Cells were plated into 96-well plates (2,000 cells/well), and treated with 10 μL CCK-8 reagent at different time-points. Varioskan Flash reader (Thermo Fisher Scientific) was used to detect the optical density at 450 nm (OD450); the results obtained were the basis for observing cell proliferation levels.

### Cell immunofluorescence assays

Cells were fixed using 4% paraformaldehyde (PFA), permeated and incubated with anti-LC3 antibody (ab192890, 1:1000, Abcam) at 4 °C overnight to complete immunofluorescent analysis. Subsequently, the above cells were stained with ﬂuorochrome-labelled secondary antibody for 1 hours and then counterstained with DAPI for 15 min. Ultimatelt, cells were observed and recorded using Olympus IX83 fluorescent microscopy. The cells containing more than three LC3-puncta dots were considered LC3-puncta-positive cells.

### Transmission electron microscope (TEM) assays

Enriched monocytes were incubated on 6-cm dishes, and stimulated with osteoclast-differentiation inducer. The preparation of cell sections, staining, and TEM analyses were carried out according to manufacturer’s protocols (Servicebio, Wuhan, Hubei, China), as described previously (Ke et al., 2019). Then, the stained sections were observed under Hitachi 7700 transmission electron microscopy (Tokyo, Japan).

### Co-immunoprecipitation (Co-ip) assays

The cell proteins were extracted with RIPA lysis and extraction buffer. Subsequently, 100 μL ice buffer was prepared to rinse the beads followed by the addition of 100 μL antibody-binding buffer, rotation of the antibody and magnetic beads, and Cleaning of beads with 200 μL buffer. The lysates and antibody-binding beads were incubated at room temperature for 1 hour and rinsed using 200 μL buffer. The beads were rinse using 20 μL of elution buffer to remove the supernatant. The lysates were prepared for Co-ip assays with BCL2 or Beclin1 antibodies (BCL2, 1:3000, 12789-1-AP, Proteintech; Beclin1, 1:3000, 11306-1-AP, Proteintech), and then observed and quantified based on Western-Blot assays with Beclin1 or BCL2 antibodies (refer to Western-blot assays).

### Reverse transcription-quantitative Polymerase Chain Reaction (RT-qPCR) assays

The total RNA was extracted and purified by the TRIzol method. cDNA Synthesis and RT-qPCR measurements were performed by standard protocols. The designed primer sequences for RT-qPCR were as following:

TRAP:

5’-GCACGCAGGAAGCAAGACAC-3’ (forward) and 5’-AGAGGCTGTGGCTGTGATTACC-3’ (reverse);

Cathepsin K (CTSK): 

5’-AGGCTGGAGTGTGGTGATATAGTC-3’ (forward) and 5’-GAGGCTGAGGCGAGAGGATTG-3’ (reverse);

Matrix metalloproteinase 9 (MMP9):

5’-AATGGCAGAAGAGATGGTTGTCAAG-3’ (forward) and 5’-ACAGGCAGGCATAAGAGGAGTG-3’ (reverse);

GAPDH:

5’-GGAGCGAGATCCCTCCAAAAT-3’ (forward) and 5’-GGCTGTTGTCATACTTCTCATGG-3’ (reverse).

In the RT-qPCR detection, the melting curve was analyzed to ensure that there was no amplification artifact. RT-qPCR was carried out using SYBR Premix Ex TaqTM kit (TakaRa, Tokyo, Japan) and ABI7500 PCR system (Applied Biosystems, ThermoFisher Scientific, MA, USA). The PCR cycle conditions were set as: 94 ℃ for10 min, 95 ℃ for 15 s, and 60 ℃ for 60 s within 40 cycles, and 3 replicate wells were set for all reactions to ensure the accuracy of the data.

### Detection of apoptosis

The apoptosis was evaluated by measuring apoptotic cell abundance using Annexin V/PI (AV/PI) staining assays. Cell were treated with corresponding treatments for 24 hours, and then staining was carried out by standard protocols. Next, the apoptotic degree was measured and quantitatively analysed using flow cytometer (BD Accuri C6 Plus, BD Biosciences). Apoptotic cells were displayed as Annexin V-positive cells.

### Statistical analysis

The one-way and two-way ANOVA were used to analyze the data after determining their normal distribution. Tukey test was used for Post-Hoc multiple comparisons of ANOVA. All experiments were performed on the basis of three replicates. Data are represented as means± SD. The threshold for P-value is set to 0.05. All statistical analyses were performed using IBM SPSS26.0 software (IBM Corp., Armonk, NY, USA).

### Ethics statement

Ethical approval for collection of human samples (Ethical Committee K2022-09-055) was provided by the Ethical Committee of Fujian provincial hospital, Fuzhou, China. Written informed consent was obtained from 5 participating probands prior to sample collection.

## Results

### Osteoclasts derived from TF- monocytes increased while those of TF+ monocytes decreased

**Figure 1-:**
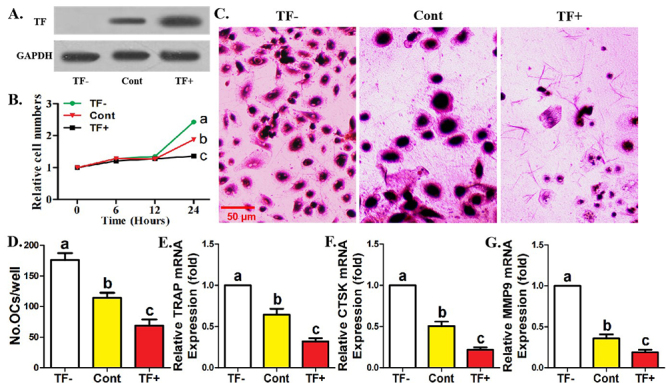
Osteoclasts derived from TF^-^ monocytes increased while those of TF^+^ monocytes decreased. **A** Western-blotting of TF among three monocytic clusters enriched by cell sorting. The relative levels of TF are expressed as the ratio of TF to GAPDH. **B** CCK-8 assays showing the proliferative ability of three monocytic clusters. **C** TRAP staining showing the capacity of three monocytic clusters to differentiate into mature osteoclasts. **D** Quantification of osteoclastogenic ability for three monocytic clusters. **E-G** Quantification of mRNA levels of osteoclastogenic genes for three monocyte clusters after osteoclastic induction. Data come from three independent repeated experiments and are represented as means± SD. The demotion in letters indicates a significant decrease with *P*<0.05. One-way ANOVA and Tukey Post-Hoc multiple comparisons were used for (**B, D-G**). Cont, control group; TF^-^, TF-negative monocyte group; TF^+^, TF-positive monocyte group.

Firstly, we elucidated the significance of TF in the osteoclastogenic potential of monocytes. As shown in Figure 1 A , the sorting efficiency of TF^-^ monocytes and TF^+^ monocytes was identified. It was observed that TF^-^ monocytes exhibited stronger activity in proliferation and differentiation towards mature osteoclasts under osteoclastic induction (Figure 1 B-D ). The results of osteoclastogenic genes showed a similar trend as cell phenotype based on TRAP staining (Figure 1 E-G ). However, TF^+^ monocytes exhibited completely opposite results to TF^-^ monocytes on all parameters (Figure 1 B-G ). The adverse effects of TF on osteoclastogenesis derived from human monocytes were revealed.

### Autophagy of TF- monocytes strengthened while that of TF+ monocytes decreased

**Figure 2 -:**
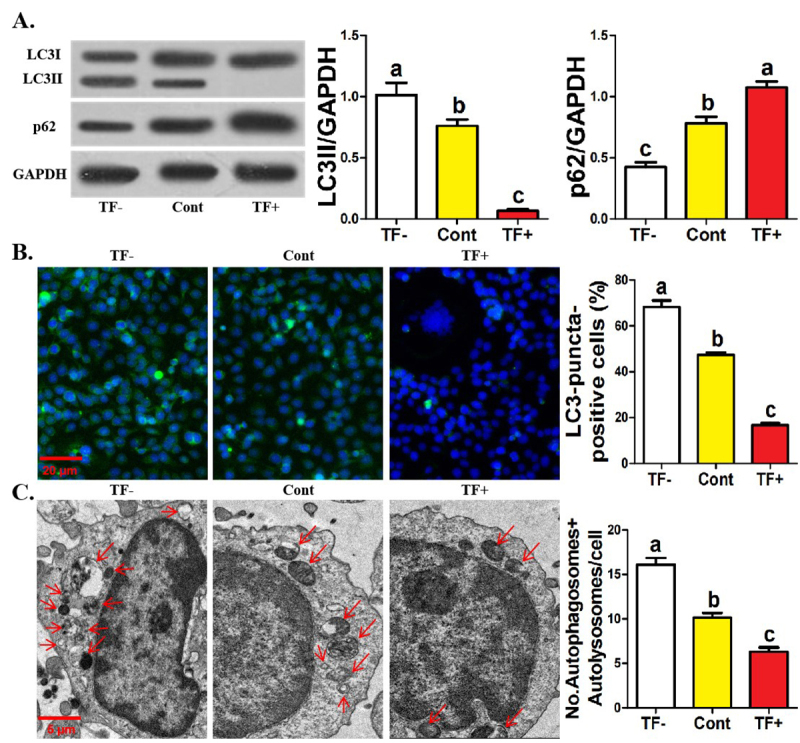
Autophagy of TF^-^ monocytes strengthened while that of TF^+^ monocytes decreased. **A** Western-blotting of LC3 and p62 among three monocytic clusters after 12 hours of osteoclastic induction. The relative levels of LC3II and p62 are expressed as the ratio of target proteins to GAPDH. **B** Immunofluorescence assays showing the formation of LC3-puncta and quantification of LC3-puncta-positive cells among three monocytic clusters after 24 hours of osteoclastic induction. **C** TEM showing the formation of autophagosomes and autolysosomes and the abundance of autophagosomes/autolysosomes for 45 cells among three monocytic clusters after 24 hours of osteoclastic induction. Data come from three independent repeated experiments and are represented as means± SD. The demotion in letters indicates a significant decrease with *P*<0.05. One-way ANOVA and Tukey Post-Hoc multiple comparisons were used for (**A-C**). Cont, control group; TF^-^, TF-negative monocyte group; TF^+^, TF-positive monocyte group.

Then, we investigated the effect of TF on the autophagic activity of monocytes within osteoclastic induction. During autophagic responses, LC3I is modified and processed by a ubiquitin-like system to form LC3II that attaches to the membrane of the autophagosomes, forming the structural protein of the autophagosomes and p62 is selectively encapsulated in the autophagosomes and subsequently degraded by proteolytic enzymes in the autolysosomes as a bridge between LC3 and polyubiquitinated proteins, indicating that LC3II protein level positively reflects autophagic activity while p62 protein level is opposite (Ichimura et al., 2008). We found that under osteoclastic induction, LC3II protein expression of TF^-^ monocytes was significantly enhanced while their p62 protein expression was significantly reduced (Figure 2 A ). In addition, under osteoclastic induction, the formation of LC3-puncta in TF^-^ monocytes significantly increased (Figure 2 B ). TEM assays also showed a significant increase in the abundance of autophagosomes and autolysosomes for TF^-^ monocytes within osteoclastic induction (Figure 2 C ). As expected, TF^+^ monocytes exhibited completely opposite results to TF^-^ monocytes on all parameters (Figure 2 A-C ). The negative regulation of TF on the autophagic activity of human monocytes within osteoclastic induction was revealed.

### BCL2-Beclin1 interaction of TF- monocytes decreased while that of TF+ monocytes strengthened

**Figure 3 -:**
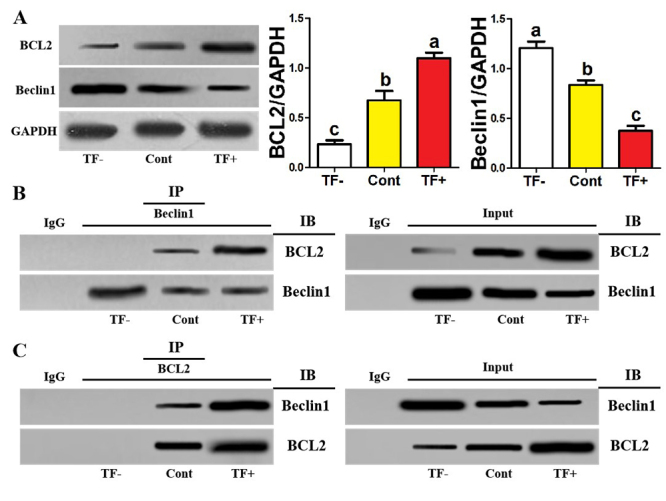
BCL2-Beclin1 interaction of TF^-^ monocytes decreased while that of TF^+^ monocytes strengthened. **A** Western-blotting of BCL2 and Beclin1 among three monocytic clusters after 12 hours of osteoclastic induction. The relative levels of BCL2 and Beclin1 are expressed as the ratio of target proteins to GAPDH. **B** Cell extracts processed in (**A**) were prepared for coimmunoprecipitation with anti-Beclin1 antibody, and then, the precipitates were observed through Western-blotting of BCL2. **C** Cell extracts processed in (**A**) were prepared for coimmunoprecipitation with BCL2 antibody, and then, the precipitates were observed through Western-blotting of Beclin1. Data come from three independent repeated experiments and are represented as means± SD. The demotion in letters indicates a significant decrease with *P*<0.05. One-way ANOVA and Tukey Post-Hoc multiple comparisons were used for (**A**). Cont, control group; TF^-^, TF-negative monocyte group; TF^+^, TF-positive monocyte group; IP, the antibody for immunoprecipitation, IB, the antibody for immunoblot.

Next, we explored the effect of TF on BCL2-Beclin1 signaling in monocytes within osteoclastic induction. We found that under osteoclastic induction, BCL2 protein expression of TF^-^ monocytes was significantly decreased while their Beclin1 protein expression was significantly enhanced (Figure 3 A ). However, BCL2 protein expression of TF^+^ monocytes significantly increased while their Beclin1 protein expression significantly decreased (Figure 3 A). Co-ip assays based on Beclin1-IP antibody showed that BCL2-Beclin1 interaction of TF^-^ monocytes was decreased while that of TF^+^ monocytes was enhanced (Figure 3 B ). Co-ip assays based on BCL2-IP antibody also showed consistent results (Figure 3 C ). In addition, the corresponding inputs exhibited the results similar to Figure 3 A  (Figure 3 C), which validates the credibility of the above results. Under osteoclastic induction, the involvement of TF in BCL2-Beclin1 signaling for human monocytes was demonstrated.

### Treatment of spautin1 recovered the parameters associated with TF- monocytes

**Figure 4 -:**
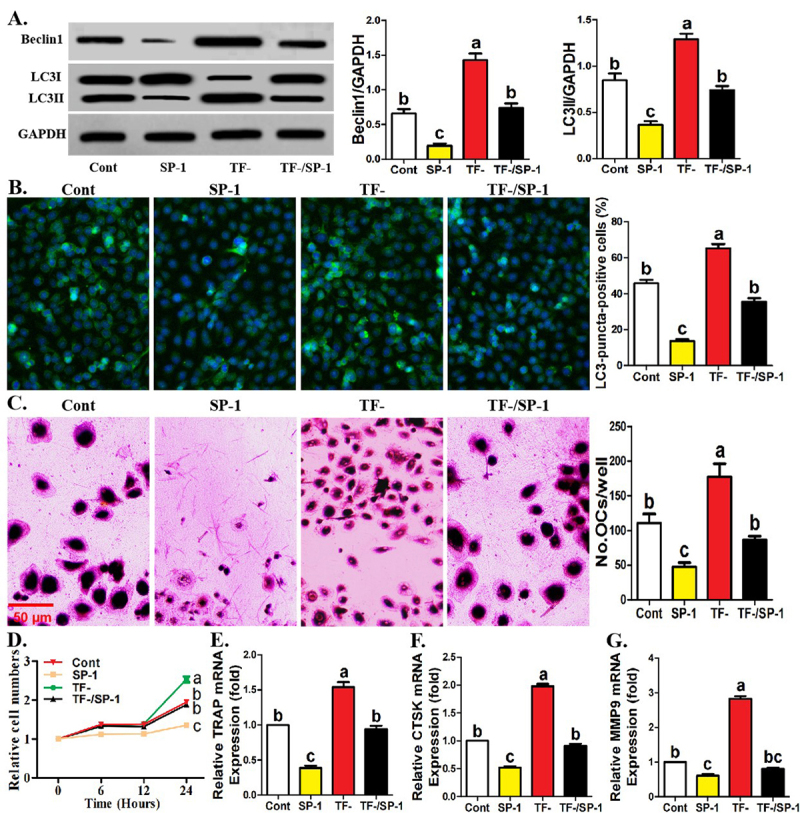
Treatment of spautin1 recovered the parameters associated with TF^-^ monocytes. **A** Western-blotting of Beclin1 and LC3 for TF^-^ and control monocytic clusters treater with or without spautin1 (10 μM) under 12 hours of osteoclastic induction. The relative levels of Beclin1 and LC3II are expressed as the ratio of target proteins to GAPDH. **B** Immunofluorescence assays showing the formation of LC3-puncta and quantification of LC3-puncta-positive cells among four monocytic clusters as described in (**A**) after 24 hours of osteoclastic induction. **C** TRAP staining showing the capacity of four monocytic clusters as described in (**A**) to differentiate into mature osteoclasts. **D** CCK-8 assays showing the proliferative ability of four monocytic clusters as described in (**A**). **E-G** Quantification of mRNA levels of osteoclastogenic genes for four monocytic clusters as described in (**A**) after osteoclastic induction. Data come from three independent repeated experiments and are represented as means± SD. The demotion in letters indicates a significant decrease with *P*<0.05, and double-letter indicates no statistical difference between given group and compared groups. Two-way ANOVA and Tukey Post-Hoc multiple comparisons were used for (**A-G**). Cont, control group; TF^-^, TF-negative monocyte group; SP-1, spautin1.

The significance of TF for BCL2-Beclin1 signaling in human monocytes was documented. We continued to explore the role of BCL2-Beclin1 signaling in TF-regulated osteoclastogenesis derived from human monocytes. As shown in Figure 4 A , under osteoclastic induction, the addition of Beclin1 inhibitor, spautin1, not only reduced the expression of Beclin1 and LC3II protein in monocytes, but also blocked the increased expression of Beclin1 and LC3II protein caused by TF negative sorting. Moreover, the administration of spautin1 inhibited LC3-puncta abundance in the control monocytes (CD14^+^ cells) and TF^-^ monocytes (Figure 4 B ). Additionally, the application of spautin1 inhibited the differentiation potential, proliferation ability and osteoclastogenic gene expression in the control and TF^-^ monocytes (Figure 4 C-G ).

### Treatment of TAT-Beclin1 or ABT-737 recovered the parameters associated with TF+ monocytes

**Figure 5 -:**
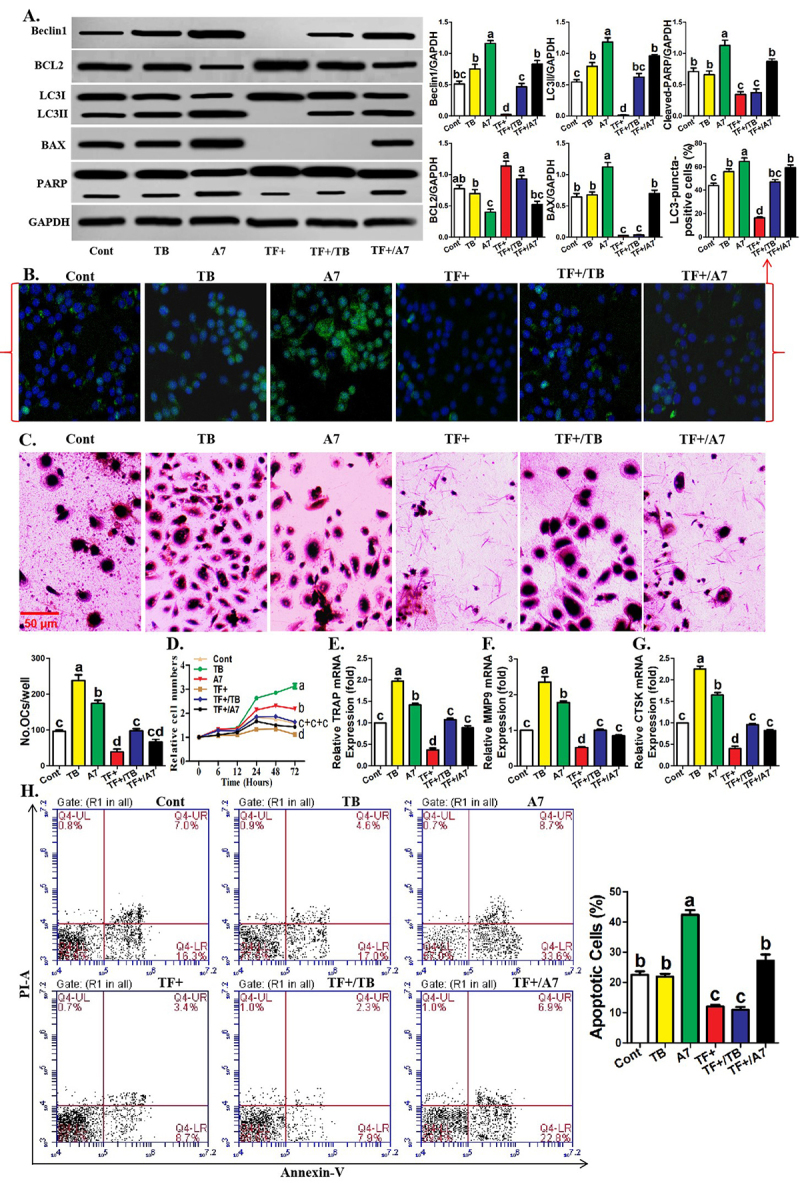
Treatment of TAT-Beclin1 or ABT-737 recovered the parameters associated with TF^+^ monocytes**. A** Western-blotting of Beclin1, BCL2, LC3, BAX and PARP for TF^-^ and control monocytic clusters treater with Tat-Beclin1 (10 μM) or ABT737 (5 μM) under 12 hours of osteoclastic induction. The relative levels of LC3II, cleaved-PARP and other proteins are expressed as the ratio of target proteins to GAPDH. **B** Immunofluorescence assays showing the formation of LC3-puncta and quantification of LC3-puncta-positive cells among six monocytic clusters as described in (**A**) after 24 hours of osteoclastic induction. **C** TRAP staining showing the capacity of six monocytic clusters as described in (**A**) to differentiate into mature osteoclasts. **D** CCK-8 assays showing the proliferative ability of six monocytic clusters as described in (**A**). **E-G** Quantification of mRNA levels of osteoclastogenic genes for six monocytic clusters as described in (**A**) after osteoclastic induction. **H** AV/PI staining assays showing apoptotic cell abundance of six monocytic clusters as described in (**A**) (Annexin V-positive cells were considered apoptotic cells). Data come from three independent repeated experiments and are represented as means± SD. The demotion in letters indicates a significant decrease with *P*<0.05, and double-letter indicates no statistical difference between given group and compared groups. Two-way ANOVA and Tukey Post-Hoc multiple comparisons were used for (**A-G**). Cont, control group; TF^+^, TF-positive monocyte group; TB, Tat-Beclin1; A7, ABT737.

Finally, we further completed the relationship between BCL2-Beclin1 signaling and TF-regulated osteoclastogenic parameters by elucidating Beclin1 pharmacological overexpression and BCL2 pharmacological inhibition. As shown in Figure 5 A , under osteoclastic induction, the addition of Beclin1-overexpressing agent (Tat-Beclin1) or BCL2 inhibitor (ABT737) directly increased Beclin1 and LC3II protein expression and reversed the increased expression of Beclin1 and LC3II protein caused by TF positive sorting in monocytes. The inhibition of BCL2 protein expression by ABT737 was also validated (Figure 5 A ). Moreover, both the two reagents increased LC3-puncta abundance in the control and TF^+^ monocytes (Figure 5 B ). Additionally, osteoclastogenic capacities from control and TF^+^ monocytes treated with both the two reagents were stronger than those from control and TF^+^ monocytes without corresponding interventions (Figure 5 C-G ). Both the two reagents increased the differentiation potential, proliferation ability and osteoclastogenic gene expression in the control and TF^+^ monocytes (Figure 5 C-G ). In addition, BCL2 can also be connected to apoptosis regulatory molecule BAX (Wei et al., 2008); therefore, we need to observe the effects of TF-based various interventions on monocytic OCP apoptosis through BAX as well as PARP cleavage level and apoptotic cell abundance that reflect the degree of apoptosis. As shown in Figure 5 A, TF^+^ monocytes showed lower expression of BAX and cleaved-PARP proteins than control monocytes, and the application of ABT737 promoted BAX and cleaved-PARP protein expression in the control and TF^+^ monocytes. Similarly, AV/PI staining showed that ABT737 administration increased apoptotic cell abundance in the control and TF^+^ monocytes and that of TF^+^ monocytes was lower. It was suggested that TF-caused BCL2 overexpression contributes to its inhibition on apoptosis and ABT737 enhances apoptotic levels through BCL2 inhibition.

## Discussion

For a long time, the prevention and treatment of pathological bone loss is a difficult problem and there has been no qualitative breakthrough in its treatment, which is attributed to the corresponding studies with the limitation on overconcerning mouse osteoclasts and neglecting human osteoclasts. As human OCPs, the mainstream development pathway of circulating monocytes is to evolve into macrophages located in various tissues to maintain the stability of the internal environment. The underlying laws behind the development of circulating monocytes into mature osteoclasts remain mysterious. The relevant factors that lead to the above development are unclear. It is worth noting that TF, which is considered an autophagy inhibitor (Xu et al., 2022; Liu et al., 2022), is widely present in monocytes (Grover and Mackman, 2018; Musgrave et al., 2023; Olivieri et al., 2023). Given the important role of autophagy in osteoclastogenesis (Montaseri et al., 2020; Wang et al., 2023), a scientific question has emerged: can TF attenuate monocytic osteoclastogenesis by inhibiting autophagy? Is the deletion of TF a reason for the development of human monocytes into mature osteoclasts? Our study confirmed for the first time the significance of TF in monocytic human osteoclastogenesis and specific molecular mechanisms.

**Figure 6 -:**
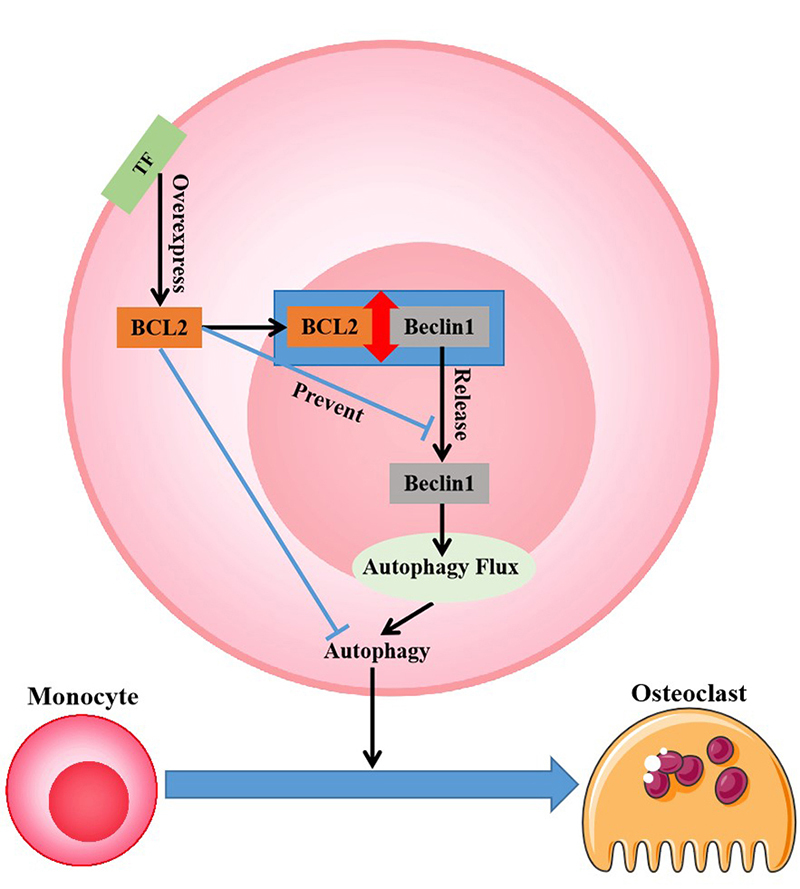
Schematic diagram showing the significance of TF-BCL2-Beclin1-autophagy signaling for the fate of human monocytes to mature osteoclasts.

Cell enrichment assays support the significance of TF in the differentiation fate of human monocytes. Unlike the obvious osteoclastogenesis caused by TF negative sorting, TF positive sorting leads to the reduction in the differentiation from monocytes towards mature osteoclasts. Monocytes in peripheral blood enter into various tissues by differentiating into macrophages, and differentiated macrophages maintain the stability of the internal environment through various biological activities such as phagocytosis, anti-inflammation and anti-infection (Yan and Horng, 2020; Brown, 2024). It is known that osteoclast maturation and macrophage activation are two independent pathways for precursor cell development (Miyamoto et al., 2001). Previous studies reported that low TF status is associated with delayed bone repair induced by diabetes status in mice, which can be attributed to the in vitro experimental evidence showing that TF significantly reduced RANKL-induced osteoclast differentiation (Ehara et al., 2021). Therefore, we infer that TF may be an unfavorable factor in monocytic human osteoclastogenesis, and its presence maintains the stability of monocytes/macrophages-dependent in vivo environment under normal conditions. A series of in vitro assays demonstrate that TF inhibits autophagic activity in monocytes during osteoclastogenesis through overexpressed BCL2. Beclin1 is an important autophagy regulator and promotes autophagy by PI3KC3 (Pattingre et al., 2005; Wei et al., 2008). As a meaningful intermediate existing in the process of autophagy activation, the stability of BCL2-Beclin1 complex depends on BCL2 expression level (Pattingre *et al*., 2005). Underexpressed BCL2 promotes the release of more Beclin1 and promotes Beclin1-related autophagy, while overexpressed BCL2 allows more BCL2 to bind to Beclin1, thereby inhibiting autophagic activity (Pattingre *et al*., 2005). Our data also indicate that the stability of BCL2-Beclin1 complex contributes to the inhibitory effect of TF on monocyte autophagy. The relevant rescue assays further support the above theory. Previous studies revealed the role of Beclin1-dependent autophagy in osteoclastogenesis (Arai et al., 2019). Furthermore, the contribution of BCL2-Beclin-1-autophagy signal transduction to osteoclastogenesis has been strongly demonstrated (Zhao et al., 2018; Ke et al., 2019). Our study revealed the significance of BCL2-Beclin1-autophagy inhibition signaling to TF-inhibited osteoclastogenesis, which further indicates that Beclin1-dependent autophagy is also an important cause for human osteoclastogenesis. It is worth noting that although BCL2 pharmacological inhibition achieved a more significant effect in repressing autophagy, it was weaker than Beclin1 pharmacological overexpression in inhibiting osteoclastogenesis. As a BCL2 inhibitor, ABT737 competitively binds to the binding site of BCL2; this causes Beclin1 and pro-apoptotic molecule, BAX, to dissociate simultaneously (Hu et al., 2024). Therefore, the application of ABT737 can increase both autophagic activity and apoptotic levels. Our data also confirm the above theory, revealing that ABT737 inhibits both Beclin1-dependent autophagy and BAX-dependent apoptosis through BCL2 inhibition. In addition, TF also inhibited BAX-dependent apoptosis by upregulating BCL2 expression. Given that the overall effect of TF on osteoclast differentiation and OCP proliferation is to promote, we believe that autophagy inhibition is still the decisive factor for TF-regulated monocytic osteoclastogenesis. Furthermore, the autophagy-regulating ability of TF enables it to ultimately achieve the effect of inhibiting osteoclastogenesis. Overall, TF-regulated human osteoclastogenic patterns can be summarized as follows: TF upregulates BCL2 to allow more BCL2 to bind to Beclin1, thereby inhibiting Beclin1-dependent autophagy and osteoclastogenic ability in monocytes. The working model regarding our study is shown in Figure 6.

In summary, We focus on monocyte development pathway and autophagy responses, igniting a new light on the biological mechanism of human osteoclastogenesis that has been less reported. We discovered that TF is an unfavorable factor for the development of human monocytes into mature osteoclasts, and BCL2-Beclin1-autophagy inhibition signaling is involved in TF-inhibited osteoclastogenesis. After release from BCL2-Beclin1 complex, Beclin1 can enter autophagy flux, thereby activating autophagy and promoting monocyte differentiation towards osteoclasts. TF can overexpress BCL2 that prevents Beclin1 release and subsequent Beclin1-dependent autophagy by binding to Beclin1; this results in the inhibitory effect of TF on monocyte autophagy and osteoclast differentiation. Therefore, we infer that TF overexpression or the weakening of any factor in the process of BCL2-Beclin1 autophagy activation signaling can be a favorable factor for decreased human osteoclastogenesis and the improvement of osteoclast-induced bone destruction.

## Data Availability

The raw data supporting the findings in this study are available upon reasonable request.
